# Exploring Caregiver Perspectives of Social and Motor Skills in Children With Autism Spectrum Disorder and the Impact on Participation

**DOI:** 10.3389/fpsyg.2020.01260

**Published:** 2020-06-19

**Authors:** P. Camila Rios, Sara M. Scharoun Benson

**Affiliations:** Department of Kinesiology, University of Windsor, Windsor, ON, Canada

**Keywords:** autism spectrum disorder, participation, social skills, motor skills, qualitative

## Abstract

Participation is a key aspect of quality of life and is essential for children’s well-being, yet children with disabilities are at risk for lower participation in social activities. For children with autism spectrum disorder (ASD), social skills may present a significant obstacle for participation in activities of daily life; however, motor skill development may also serve an important contributing factor. Nevertheless, the link between social and motor skills in children with ASD is not fully understood. The current research implemented semistructured interviews to garner descriptive insights from caregivers (*N* = 17) into the social and motor skills of 5- to 9-year-old children with ASD and the impact on participation in social activities. A constant comparative method was used to generate a coherent and thematic representation of caregivers’ experiences. Thematic analysis revealed core consistencies in three areas: (1) caregivers viewed participation differently than their children; (2) participation levels of children with ASD are context specific; (3) challenges with social skills were perceived to present a greater obstacle to participation than motor skills. Overall, the notion that ASD is a heterogeneous disorder was made very apparent. Although caregivers believe there to be immense value in current treatment and intervention options, the availability and access to such options was a major barrier. The effectiveness of intervention programming designed to increase participation is contingent on understanding factors that affect participation. Implications concerning caregivers’ perspectives are discussed.

## Introduction

Children are socially occupied beings living in a social world (Lawlor, 2003); however, because of various individual and environmental factors, children with disabilities are at risk for lower participation in social activities than their typically developing peers (Law and King, 2000; Kaljača et al., 2019). Autism spectrum disorder (ASD) is one of the most common neurodevelopmental disorders. In Canada, it is estimated one out of every 66 individuals between the ages of 5 and 17 years have a diagnosis of ASD (Government of Canada, 2016). A report from the Centers for Disease Control and Prevention’s (CDC’s) Autism and Developmental Disabilities Monitoring Network in the United States describes an increase from one in 68 children aged 8 years during 2010–2012 to one in 59 children in 2014 (Bajo et al., 2018). While difficulties with social communication and social interaction are considered defining features of ASD, characteristics fall on a spectrum and present differently among individuals. Expressions range from having significant interference in everyday life to behaviors that are often difficult to differentiate from typically developing peers (American Psychiatric Association, 2013).

Atypical social development, characterized by language deficits and problems with pragmatic aspects of language, is a defining characteristic of ASD (American Psychiatric Association, 2013). Challenges coordinating speech with eye contact, demonstrating a genuine interest in others’ thoughts and opinions, and the ability to acknowledge when to start and end a conversation are commonly reported (Mundy et al., 1990; Dawson et al., 2004). In addition to language and social challenges, stereotyped, restricted, or repetitive behavior patterns, a necessity for routines and rigidity and limited interests are also cardinal features of ASD (American Psychiatric Association, 2013).

While diagnostic features manifest differently and present unique challenges for each child, it is generally understood that characteristics of ASD have a significant impact on socialization and involvement within all aspects of life. Decreased participation levels and less variety in activities are reported among children with ASD compared to both typically developing children and children with other developmental disabilities (Shattuck et al., 2011; Askari et al., 2014; Little et al., 2014; Taheri et al., 2016; Egilson et al., 2018). For school-aged children and adolescents with ASD, participation levels are lower at home and school and much lower in the community (Simpson et al., 2018, 2019; Lamash et al., 2020). Participation in unstructured and recreational activities, hobbies, after-school peer socialization, and physical activities (e.g., sports teams and clubs) are key areas of concern (Hilton et al., 2008; Hochhauser and Engel-Yeger, 2010; Potvin et al., 2013; Little et al., 2014; Scharoun et al., 2017; Egilson et al., 2018). While adolescents with ASD spend 62% more time with screen-based media (e.g., television, video, and computer games) compared to other activities, few children participate in socially interactive games or social media activities (Mazurek and Wenstrup, 2013). Parents most desire a decrease in participation in screen-based activities (Simpson et al., 2018). This is not surprising; preoccupation is a clinically significant problem for many children with ASD, and growing evidence indicates excessive use can have detrimental effects on academic performance, social interaction, and physical activity (Mazurek and Wenstrup, 2013). A decline in participation in social and physical activities (e.g., sport and exercise) is documented with age, with sedentary behaviors becoming more common toward adolescence (Ketcheson et al., 2017; Ratcliff et al., 2018; Simpson et al., 2019).

Although social skills are typically regarded as a significant concern for children with ASD, motor deficits have also been acknowledged. Fine and gross motor challenges and issues with balance and coordination have been reported (Fournier et al., 2010; Gowen and Hamilton, 2013). It is estimated that 79% of children have some sort of motor difficulty (Pusponegoro et al., 2016). Retrospective video studies report the emergence of motor delays early in life (Teitelbaum et al., 1998), and the rate of development of motor skills for children with ASD is slower than for their typically developing peers. Thus, the gap in respective motor skills appears to widen over time (e.g., Lloyd et al., 2013). Despite evidence that children with ASD have motor deficits and that these challenges are evident early in life, early intervention is generally focused on improving social skills (MacDonald et al., 2014). Only recently has research interest been directed toward motor intervention (e.g., Bishop and Pangelinan, 2018).

A review of motor abilities in ASD posted the question of “how motor difficulties relate to social difficulties – are they independent or do underlying motor issues cause social characteristics?” (Gowen and Hamilton, 2013, p. 340). As movement facilitates opportunities to engage in social activities and develop social relationships, developing motor skills establishes an important foundation for social development (Leonard and Hill, 2014). Evidence indicates children with ASD who have weaker motor abilities often face greater challenges in social domains as well (MacDonald et al., 2014; Scharoun et al., 2015; Ohara et al., 2020). It has also been argued that motor challenges may underlie the significant reduction in the variety of activities children with ASD participate in, because of lowered self-esteem and perceived skill level (Pusponegoro et al., 2016).

Overall, despite research showing that social development and motor development are interdependent (Leonard and Hill, 2014) and that motor competence contributes to participation (Pusponegoro et al., 2016), differences in participation levels between children with ASD and typically developing children are primarily attributed to challenges with social skills (Little et al., 2014). The bidirectional impact of social and motor domains on participation among children with ASD has only recently become a topic of interest. Holloway and Long (2019) published a perspective piece on the topic, stating “we use ASD as a model disorder to demonstrate the interrelationships between motor skill performance and the social interaction component of participation because it has not been examined previously in the literature and because of the potential importance to this population” (p. 765).

There is a clear gap in the literature regarding how motor challenges, and the interplay between motor and social deficits, affect daily participation among children with ASD at home, school, and in the community. Substantial literature has examined the extent of participation, and considerable work has focused on developing and implementing interventions to increase participation; however, individual perspectives and experiences are not typically considered in the participation literature (Martin Ginis et al., 2017). As caregiver appraisal of child development is a key component of pediatric evaluation (Majnemer and Rosenblatt, 1994), the current research aimed to garner descriptive insight of caregivers’ perspectives of their children’s motor and social skills and how they impact participation. More specifically, we used a qualitative method (Patton, 2014) to delineate whether caregivers were concerned with their child’s motor functioning as a factor influencing participation, challenges with social skills were the primary focus, or an interrelationship between social and motor skills was recognized.

## Materials and Methods

### Procedures

After receiving clearance from the institutional research ethics board, the second author contacted child development centers and organizations across Southwestern Ontario to request permission to purposefully recruit participants. The researcher had no prior roles or relationships with any of the organizations or potential participants. Site-specific approvals were forwarded to the institutional research ethics board noting which specific recruitment method (e.g., posters, flyers, Listserv) would be employed. To ensure confidentiality, the research team did not have access to client contact information; therefore, recruitment information was shared with clients who met the inclusion/exclusion criteria (i.e., families of 5- to 9-year-old children with a diagnosis of ASD) on behalf of the researchers. Families who were interested in the research contacted the second author to obtain additional information and schedule data collection.

This study is part of a larger mixed-methods project. Families also participated in the quantitative portion of the project, which is examining relationships between motor function, social function, and participation among children with ASD. In qualitative research, both “participants’ and researchers’ interpretations of phenomena are taken into account in the process of analysis” (Pietkiewicz and Smith, 2014, p. 7). As the objective of the current research goal was to authentically represent caregiver’s perspectives, the quantitative data were not reviewed prior to completing the qualitative phase of this project to help prevent the researchers’ interpretations from being guided by quantitative assessments.

### Participants

The current study (*N* = 17) included 14 mothers, 2 fathers, and 1 grandmother (aged 32–75 years, mean = 42.18 ± 9.75 years) of children between the ages 5–9 years (mean = 7.12 ± 1.17 years; 14 male and 3 female). No other sociodemographic information was collected from caregivers, which can be considered a limitation of this research. To ensure interviewer consistency, the second author collected all data through a combination of in-laboratory (*n* = 2), at-home (*n* = 10), and phone-based (*n* = 5) interviews, according to participant preference. As a thank-you for participating in the interview, a $10 gift card to a local bookstore was provided.

### Semistructured Interviews

Semistructured interviews were conducted to garner descriptive insight of caregivers’ perspectives of their children with ASD. A semistructured interview guide was used for data collection, which occurred between September 2018 and January 2019. Interviews, which ranged between 13 and 38 min, were digitally audio recorded using a SONY ICD-PX370 recorder. To improve the depth and richness of participants’ responses, each interview commenced with background information about the child (i.e., age, sex) and experiences obtaining/receiving a diagnosis of ASD (e.g., “When was your child diagnosed?” “How long prior to diagnosis were signs/symptoms noticed?” “What made you initially seek diagnosis?”). Participants were also asked questions about current and past involvement in therapies and/or interventions (e.g., “Is your child accessing or involved in any therapies/interventions” “How long has your child been involved?” “What is your role in this routine?”). These questions were designed to build rapport and encourage a conversational flow.

We then asked participants to define participation in their own words to elicit general thoughts on the topic. Subsequently, the *International Classification of Functioning, Disability, and Health* definition of “involvement in a life situation” (World Health Organization [WHO], 2001, p. 10) was provided to establish a consensus and encourage contextually rich descriptions of the quality and quantity of their child’s participation. Based on these initial responses, we explored a typical day (school and weekend) for each child (e.g., “Can you please describe a typical day for your child?” “What activities does your child participate in?” “How would you describe participation levels?” “What environmental factors influence your child’s participation?” “Would you like to see your child participate in other activities?” “Has participation changed at all over time?”). Follow-up questions (e.g., “Can you please describe your child’s [social/motor] skills?” “Do your child’s [social/motor] skills influence participation?”) and probes (e.g., activity limitations, participation restrictions, changes over time) allowed us to garner perspectives of specific aspects of social (e.g., awareness, cognitive, communication, motivation) and motor function (e.g., fine motor precision/integration, manual dexterity, bilateral coordination, balance, speed/agility, strength), the interaction between social and motor skills, and influence on participation over the course of the child’s life. As semistructured interviews offer the flexibility to explore participant experiences, each interview did cover the same key areas of interest; however, the order of questions varied based on the nature of participant responses. At the end of each interview, participants were asked “if there is anything that we missed or anything you would like to add that you feel will help us better understand your personal experience.”

### Data Analysis

The first author transcribed interviews verbatim; however, identifying information and language errors (e.g., like and um) were removed to help with readability and clarity of ideas within the article. Interviews were thematically analyzed by the second author using NVivo12 (QSR International, Melbourne, Australia). Transcripts were examined to garner a sense of the data and enable an open-coding process. The following questions (Srivastava and Hopwood, 2009, p. 78) were used to guide data analysis: (1) “What are the data telling me?” (e.g., What are the interview data from caregivers telling us about their children’s motor and social skills, and how do they impact participation?); (2) “What is it I want to know?” (e.g., Identifying whether caregivers are concerned with their child’s motor functioning as a factor influencing participation, or if challenges with social skills are the primary focus; likewise, understanding whether caregivers recognize the bidirectional relationship between motor and social skills, and combined impact on participation); and (3) “What is the dialectical relationship between what the data are telling me and what I want to know?” (e.g., What do interview data from caregivers tell us?). A constant comparative method was employed, whereby data collection, transcription, and analysis continued until saturation of themes and of relationships among them was achieved (Saunders et al., 2018). More specifically, guiding analysis questions were repeated until a more refined and explicit picture of caregivers’ perceptions was revealed (Srivastava and Hopwood, 2009). At this point, the first author independently reviewed transcripts to ensure accuracy (i.e., that codes and themes accurately reflected and appropriately grouped the topics emerging from conversations). Researchers met to discuss any potential discrepancies, and no issues were identified; however, if disagreement had been raised, a third coder would have been included, until agreement was reached.

## Results

Thematic analysis revealed core consistencies in the data: (1) caregivers viewed participation differently than their children; (2) participation levels of children with ASD are context specific; (3) challenges with social skills were perceived to present a greater obstacle to participation than motor skills. The notion that ASD is a heterogeneous disorder was made very apparent. For example:

It’s not a joke, but it kind of sounds funny when you say it, you know, that when you meet one child with autism, you’ve met one child with autism because there’s no such thing as a standard. Like, the *DSM* exists but even within that there are so many variants (P11).

The following outlines overarching themes and highlights respective subthemes that emerged from the interviews, while describing similarities and differences in experiences. Representative quotations are included to help illustrate caregivers’ perspectives.

### Caregivers Viewed Participation Differently Than Their Children

Participants’ descriptions of participation were concurrent with societal views, ranging from almost verbatim accounts of the *International Classification of Functioning, Disability, and Health* definition (i.e., “involvement in a life situation;” World Health Organization [WHO], 2001, p. 10), to more elaborate descriptions:

I think of kids who are actively engaged back and forth with other people, or in the community are able to move toward independence, where they would be able to go out and actively seek the library or… go to the store, and those sorts of things. That’s what I think of as, participation… he’s participating fully 100% from his view because he doesn’t care to participate, but from what we would consider to be the norm, he’s not there for sure” (P8).

Generally, caregivers acknowledged the potential disconnect between personal perspectives of participation and how their children with ASD may view and thus experience participation.

Children were described as having varied thresholds for participation in group and social activities:

I mean it’s always a great benefit to try and get him involved, but you know with these kids, we generally try to let them go at their own pace. You only want to push them so hard; you don’t want them to be completely turned off (P1).

One mother described how grateful she was that her child enjoyed social activities and sought out opportunities to engage with other children: “It’s very rare… I mean he’s a social rock star” (P2). Some of the children were willing to try group activities, but not participate for the entire duration: “After 10 min, he’s like okay, that’s enough, that was fun, that fills my bucket, and it’s over for me” (P8). Others had a general preference for individual or one-on-one activities and thus avoid or resist group activities all together. For example, one caregiver expressed, with reference to preferred activities: “I mean, his favorite, I would have to say, anything he can do by himself” (P16).

Related to the threshold, caregivers highlighted factors that generally influence participation. Managing the physical and sensory elements of the environment was of utmost importance: “If there’s too much going on, it’s harder to get him engaged. Just all the noises. If there’s too much noise, or there’s too many lights or things that will distract him, that makes it difficult” (P9). Likewise, caregivers expressed the need to ensure structure, consistency, and predictability to promote success: “If they veer from any sort of plan it’s a no” (P10). Descriptions also centered around children’s motivation, confidence, and competence: “if he feels like, he can’t do something he won’t participate” (P1). Some caregivers discussed a complete lack of social motivation. For others, lower levels of perceived motor competence resulted in decreased self-confidence, which ultimately discouraged participation. Some children were thus described as unwilling to try new activities – or would try, but, after failing once, not trying again.

An important point of consideration was the notion of whom children participate with. Whereas some children preferred to engage with peers of the same age, others were better able to connect with younger children. For example, one caregiver expressed: “…they are significantly younger, but he tends to do very well with them because they play at his level, kind of thing” (P11). Generally, tendencies reflected the desire for interaction with individuals at or around the same perceived “level” of functioning, or what was perceived by the child as the same or most similar “level” of functioning. This often presented quite the challenge:

It’s hard to find other kids that are a similar level that he is, and then his patience with people is very little. He’s very much like, “Well, this kid doesn’t even talk,” and well, he doesn’t talk, but they’re trying to teach him, he’s not good at it. He gets discouraged by other kids really easily, and I think that limits his participation (P8).

Some children with ASD did not fare well with their peers; therefore, caregivers expressed a preference for interaction with adults.

### Participation Levels Are Context Specific

Whereas the previous theme depicted how children with ASD view participation, data also revealed differences in children’s participation levels at home, school, and in the community. While generally described as lower than expected, caregiver agreed that the level of participation “depends upon the situation” (P11). With the greatest participation at home and least in the community, caregivers described how perceived comfort levels both promote and restrict participation: “At home, he would participate if we were having a family game, doing something as a family, he would participate. But home is his comfort. Anything outside the home would be the least amount possible” (P13). The discussions surrounded the comfort levels of not only the child, but also the broader family unit. Some families were eager to explore new activities and social environments: “you know we’re not the type of parents that sit back and go like whatever… we try to get him involved in everything that is going on” (P1). In comparison, others were more hesitant to try things outside their comfort zones. As a result, some caregivers described a preference to engage in programming designed exclusively for children with special needs or ASD, whereas others felt it was extremely important to enroll their children with typically developing peers as a foundation for the future: “But at the same time I’m like, at some point he’s gotta do stuff, he’s gonna have to go to work, he’s going to have to do those things” (P8). Children represented in the current study were quite young, with an average age of approximately 7 years, yet caregivers were cognizant of how decisions regarding activity selection, and participation opportunities, in the short-term would affect their children in the long run.

Considering the young age range of child participants, many had recently transitioned into school; therefore, school participation was a key topic of discussion. At the time data were collected, all children were enrolled full-time at publicly funded elementary schools in Ontario, Canada; however, participants acknowledged periods where children were attending only part-time and/or required extra days off, primarily due to behavioral issues. At school, educational assistants (EAs) help to promote participation in regular classrooms, while also facilitating the support necessary for each Individual Education Plan (Ontario Ministry of Education, 2004). As such, children were described as spending varying amounts of time in vs. outside the classroom and engaging with classmates vs. the EA. For example:

It’s a lot of 1-on-1. But, he’s getting better with trying to figure out how to do things as a group… Now he can go out for recess with the other children, and yes, he still has an EA, but the EA can stand back and let him try to play with the other kids (P16).

Central to all discussions was appreciation for a supportive school environment. Caregivers discussed cultures of acceptance and inclusive climates: “At school, they put up the autism flag last year. And he was the one that raised it” (P15). For most of the families, school was described as the place for regular and consistent support. In addition to full-time teachers and EAs, caregivers described outside specialists (e.g., occupational and physical therapists, speech/language pathologists) visiting classrooms to address specific concerns (e.g., behavioral issues, motor/social/communication skills), with positive transfer from school to both home and community-based participation.

Notwithstanding the previous, the need for additional support outside of school was expressed. Acknowledging the immense value of treatment and intervention options, availability and access were described as a major barrier:

“Everything right now is on hold. We’re on a waiting list. He’s got an iPad at school, but I don’t know who programs or runs that. That doesn’t come home. Yeah, we’re on 3 different waiting lists for speech, for special services at home, for Respite for him, and the [provincial organization] whatever they’re supposed to do. So, we’re winging it” (P5).

Wait lists and children aging out of eligibility before being able to access services were a key area of concern. Caregivers described the reliance on short bouts of therapy and/or interventions to help guide individual and family oriented practices:

“We try. We try new things. I’ll get an idea from somewhere and be like, you know, we’re not going to wait for this to come to therapy, let’s try something different… I just – I can’t afford – I don’t have the money myself to pay for anything, and even – even with the ABA and the funding option, because they don’t give you what it actually costs to go private, they only give you a certain amount of money. So, I can’t do it; I have to wait for government funding” (P16).

For some families, community activities (e.g., sports, clubs, etc.) supplemented or complemented school-based programming. For others, activities were viewed as extracurricular activities separate from therapies and/or interventions. Regardless of intent, caregivers admitted they were doing what was feasible with the time and resources available to their families, but would like to see their children participate more to encourage continued development in all areas, including social and motor skills.

### Challenges With Social Skills Were Perceived to Present a Greater Obstacle to Participation Than Motor Skills

Many caregivers spoke to their children’s strengths in gross motor proficiency; therefore, such skills were not of major concern. Activities such as jumping on the trampoline, running, climbing, and other “big” movements were favored and often described as an outlet for children. That said, caregivers acknowledged that motor skills were indeed delayed in comparison to typically developing children, describing specific challenges with balance and coordination: “it’s kind of clunky, it’s not as fluid as the kids his age” (P11). Another expressed, “He’s really clumsy; he bumps into walls” (P5). In contrast to gross motor skills, caregivers agreed fine motor skills present daily challenges for children. The two aspects described most frequently were self-care (e.g., getting dressed) and classroom work (e.g., writing, cutting with scissors). As one caregiver described: “I don’t worry about him and mobility until it comes to the fine things. You know, I still have to brush his teeth. Wash his hair. Yeah, there’s stuff that kids his age can do that he just – he’s not there yet” (P16). Another expressed:

“He still has issues with writing, he’s getting it, but it’s definitely been a very long process. I, as a parent, can make out what he writes most of the time but new people, they definitely have to question, you know, what he’s written and that sort of stuff, so still can’t stay in the lines when it comes to coloring and all that fun stuff” (P1).

In conjunction with descriptions of challenges with fine motor skills, caregivers described challenges related to cognitive elements of motor control: “you know the part of her motor abilities that are more mental, that you can’t see” (P3). That said, parents acknowledged improvement with practice: “They’re getting better. The more he practices, the better he is getting” (P14).

Although many important points about motor function were raised, caregivers were generally more concerned with and thus emphasized to a greater extent the influence of social challenges in daily life. Caregivers emphasized that their children were either hypoaware or hyperaware of others. Hyperawareness of others’ non-verbal cues, attitudes, and expressions often resulted in social hesitation and avoidance; therefore, caregivers emphasized the need to prepare children in advance for new activities and upcoming events:

“He’s somewhat hesitant, but once you kind of get to his level, I mean, you saw. We’ve been warming up for the last 2 weeks that, you know, someone was coming for science, and that was exciting for him, but if you’d just shown up at the front door he would have been, you know, behind a chair, underneath an adult, not talking, wouldn’t even look at you” (P11).

Likewise, once a relationship was severed, it was very difficult to mend. In contrast, children who were described as hypoaware often appeared more social on the surface, albeit often lacking the skills to interpret, reciprocate, and respond to social behaviors: “He just kind of barrels into a house or barrels into a situation, and it’s like come what may. You know, if it’s good, it’s good, if it’s bad, it’s bad” (P12). With this lack of understanding, children were often unaware of teasing or mistakenly perceived remarks or actions as offensive.

Communication and self-expression were also a significant consideration. For caregivers of verbal children, conversation was often interpreted at face value, and remarks were often inappropriate, therefore presenting obstacles for participation in group and social activities:

“In hockey it’s good because they have mouth guards. So, he would chirp and scream at other kids and get upset, but people can’t hear him cuz the mouth guards, right? So, that’s a really nuance thing, but it was a saving grace. Whereas anything like basketball, soccer, where they can hear him, he’ll say things to kids that are very discouraging. So, it’s hard because you want him to be physically able to participate in things, but, the social aspect of losing, or his perception of the rules, right? It’s not a good match. It’s very hard to be able to find other activities that he would be able to participate in” (P8).

In comparison, with people generally unwilling or unsure of how to engage with non-verbal children: “He’s non-verbal… He does love playing with people, but the people that we know, they don’t stick around when there’s no communication. They look around and go; maybe we should just leave him alone because it’s better that way” (P10). Caregivers empathized with how frustrating it must be to lack the ability to express oneself and contribute to conversation in a typical manner and therefore understood the impact on participation levels.

## Discussion

Overall, the current study aimed to explore caregivers’ perspectives of social and motor skills of their children with ASD and how they influence participation. More specifically, we sought to determine whether caregivers were primarily concerned with their child’s motor skills, social skills, or recognized the bidirectional relationship between motor and social skills. As our semistructured interview guide included a range of open discussion points, caregivers had the freedom to elaborate on their own experiences. We thus garnered descriptive insight into unique profiles of children and their engagement in activities of daily life. We identified three overarching themes from the data: (1) caregivers viewed participation differently than their children; (2) participation levels of children with ASD are context specific; (3) challenges with social skills were perceived to present a greater obstacle to participation than motor skills. Although each theme was largely cohesive, caregivers’ experiences with their children did indeed reflect the spectrum that is inherent to autism. As recent literature has focused on elucidating this heterogeneity (e.g., Lombardo et al., 2019), the present findings offer important detail into a combination of topics that, until recently, had yet to be examined together (Holloway and Long, 2019). The following will elaborate upon each of these themes, in the context of existing literature.

### Caregivers Viewed Participation Differently Than Their Children

Caregivers acknowledged that their children’s ideas around participation may indeed be different from their own perceptions and societal norms. As such, the assumption that more participation is better (Liptak et al., 2011) may not necessarily apply for children with ASD. When reflecting on participation levels, caregivers also discussed concern about their children’s futures (e.g., independent living, getting a job). Such worries may ultimately place pressure on what caregivers and their children view as appropriate participation. Considering perspectives from the current research, it can be suggested that caregivers of children with ASD may also view participation differently than caregivers of children who are typically developing.

Participation has been described by individuals with disabilities as “a complex and multidimensional construct. There is no gold standard for ideal or optimal participation” (Hammel et al., 2008, p. 1454). Substantial literature has examined the extent of participation, and considerable work has focused on developing and implementing interventions to increase participation. Yet, as the notion of participation is objective and performance-based, individual perspectives and experiences are not typically considered (Martin Ginis et al., 2017). Martin Ginis et al. (2017) emphasized the need to broaden the conceptualization of participation to consider subjective aspects; however, their work is specific to persons with physical disabilities. As evidenced in the current findings, there are indeed unique demands that can hinder or facilitate participation for children with ASD compared with typically developing children, and children with other disabilities for that matter.

Recent literature (Arnell et al., 2018) revealed five specific aspects (freedom of choice, competence and confidence, motivation, adjustment to external demands, and predictability) that must be met for high-functioning adolescents (ages 12–16 years) with ASD to participate in physical activities. Whereas the previous findings were derived from the perspectives of adolescents with ASD, the current research offers caregivers’ perspectives of their children. Consistent with the notion of conditional participation (Arnell et al., 2018), a threshold for participation and specific factors supporting participation were revealed. Features of the environment were described as influential moderating factors; in particular, managing sensory (e.g., noise, lighting) and physical features (e.g., space definition and/or organization) were the most reported barriers to social and physical activities in the current study (Ben-Sasson et al., 2009; Lamash et al., 2020). As a result, caregivers reported more familiar, consistent, and predictable activities were less challenging to navigate and thus facilitated participation for their children.

Caregivers also discussed the role of motivation, competence, and confidence (Arnell et al., 2018) in the threshold for participation. Whereas social motivation was limited for some children, others had lower levels of perceived motor competence, and vice versa. Regardless of the combination, there was general agreement that motivation and competence in social/motor domains were linked to confidence, which ultimately encouraged or discouraged participation. Related research has similarly demonstrated that enjoyment and intensity of involvement among children with and without disabilities can be predicted by levels of competence (King et al., 2013).

Connected with this were caregivers’ descriptions of whom children participate with. Although a range of age preferences were discussed, the consensus was that children with ASD strive to engage with individuals whom they perceive to be at the same “level.” As parents agreed this presents quite the obstacle in daily life, it is not surprising that anxiety and fear related to peer relationships are prevalent in ASD (Brewster and Coleyshaw, 2011; Chen et al., 2016). As a result, less interaction has been shown to make physical activities more tolerable and enjoyable for children with ASD (Potvin et al., 2013; Ayvazoglu et al., 2015; Stanish et al., 2015). Overall, when considering thresholds for participation, factors influencing participation, and whom children prefer to participate with, caregivers agreed it would be great to have children participate more in group activities, such as team sports (Simpson et al., 2018). Nonetheless, caregivers also acknowledged that their children may not want to participate in such activities or already see themselves as participating, albeit in their own way.

### Participation Levels of Children With ASD Are Context Specific

Related to the notion of different perspectives of participation, caregivers described varying levels of participant among their children with ASD at home, school, and in the community. Consistent with quantitative reports of participation frequency and involvement (Simpson et al., 2018; Lamash et al., 2020), caregivers discussed participation levels as lower than expected based on societal norms and observations of typically developing children. Of specific emphasis was the notion of context-specific participation levels, such that highest levels of participation were observed at home, relatively lower levels in school, and lowest levels in the community. Consistent with previous questionnaire-based work (Lamash et al., 2020), children who were described as facing greater challenges did not often engage in community-based activities. While individual activities were most common in all contexts, group activities typically included family members and were most often described in the comforts of the family home. The need to find balance was described by caregivers, while also acknowledging the long-term implications of encouraging participation or allowing children to guide participation levels and activity selection in the short term.

While research has reported both stable patterns and decreases in participation over time (Ratcliff et al., 2018; Simpson et al., 2019), caregivers in the current study generally described improvements in participation levels and variety of activities with age. It is important to acknowledge that children represented in this study were relatively young (mean age = 7.12 ± 1.17 years). School entry and a supportive school environment were central to discussions of improvement, with positive transfer from school to all aspects of life. While caregivers generally described the importance of school-based programming, some also expressed the reliance on such supports. Short bouts of therapy, waitlists, and aging-out before accessing services were key areas of concerns, especially for those who were not in the financial situation to fund private therapies. Consistent with numerous reports indicating the costs associated with raising a child with ASD (e.g., Cidav et al., 2012; Rogge and Janssen, 2019), caregivers expressed the need for additional resources and services to introduce new activities, encourage increased participation, and ultimately contribute to improved social and motor skills.

### Challenges With Social Skills Were Perceived to Present a Greater Obstacle to Participation Than Motor Skills

Concurrent with a recent perspective piece (Holloway and Long, 2019), children described by their caregivers as having more proficient motor and/or social skills were generally considered to be more capable and willing to participate in group, social, and physical activities. In comparison, children described as having lower functional capabilities in social and motor domains were considered to have greater participation restrictions and activity limitations. Adding to the literature, caregivers generally agreed that social skills are a much greater barrier for participation; therefore, motor skills serve a secondary role.

When describing motor function, caregivers were primarily concerned with fine motor challenges that impact their children’s self-care and school performance (Scharoun et al., 2015). Strengths in gross compared to fine motor proficiency were discussed; yet, caregivers also acknowledged issues with balance, coordination, and cognitive elements (e.g., motor planning) that underlie motor control (Gowen and Hamilton, 2013; Scharoun and Bryden, 2016). A recent systematic review (Ohara et al., 2020) revealed specific subdomains of fine (i.e., manual dexterity) and gross (i.e., object control/aiming and catching) motor skills were most likely to be associated to social skills in ASD. However, a greater relationship is noted between fine motor skills and social skills, overall. Only recently has early intervention in ASD been geared toward motor function (Bishop and Pangelinan, 2018) in stark contrast to the historical focus on social domains (MacDonald et al., 2014). That said, many caregivers discussed improvements in their children’s motor and social skills as a result of family guided practice and professional intervention.

## Conclusion

Overall, the current research adds descriptive findings to complement a recent perspective piece on the interdependence of social and motor skills and impact on participation (Holloway and Long, 2019). Three overarching themes that emerged from semistructured interviews helped to discern a disconnect in caregiver and child perceptions of participation, identify that participation levels are context specific, and elucidate that caregivers weigh their children’s social skills as a greater barrier to participation than their motor skills. Substantial literature has examined the extent of participation, and considerable work has focused on developing and implementing interventions to increase participation. Although the potential for autism-specific participation patterns has been acknowledged (Simpson et al., 2018), experiential aspects of participation (Martin Ginis et al., 2017; Arnell et al., 2018) must be considered. As this researcher offers the perspectives of caregivers, those of children with ASD must also be explored. Overall, the effectiveness of intervention programming designed to address participation is contingent on understanding factors that affect participation. It is thus essential to consider what is meaningful participation for children with ASD.

## Data Availability Statement

The datasets generated for this article will not be made publicly available because ethics approval was granted to share representative quotes from thematic analysis, not the full interviews. Requests to access the datasets should be directed to the corresponding author.

## Ethics Statement

The studies involving human participants were reviewed and approved by the University of Windsor REB. The patients/participants provided their written informed consent to participate in this study.

## Author Contributions

SS was responsible for the study design, participant recruitment, data collection, thematic analysis, interpretation of results, and preparing the manuscript for submission. PR transcribed all the qualitative interviews, and was involved with thematic analysis, interpretation of results, and preparing the manuscript for submission. All authors contributed to the article and approved the submitted version.

## Conflict of Interest

The authors declare that the research was conducted in the absence of any commercial or financial relationships that could be construed as a potential conflict of interest.
